# Association between food environments and fetal growth in pregnant Brazilian women

**DOI:** 10.1186/s12884-023-05947-1

**Published:** 2023-09-13

**Authors:** Audêncio Victor, Ana Raquel Manuel Gotine, Ila R. Falcão, Andrêa J. F. Ferreira, Renzo Flores-Ortiz, Sancho Pedro Xavier, Melsequisete Daniel Vasco, Natanael de Jesus Silva, Manuel Mahoche, Osiyallê Akanni Silva Rodrigues, Rita de Cássia Ribeiro, Patrícia H. Rondó, Maurício L. Barreto

**Affiliations:** 1 Faculdade de Saúde Pública- USP, School of Public Health, University of São Paulo (USP), Avenida Doutor Arnaldo, 715, São Paulo, São Paulo 01246904 Brazil; 2 Department of Nutrition, Ministry of Health of Mozambique, Maputo, Mozambique; 3 Present Address: Iyaleta - Research, Science and Humanities, Salvador, Bahia Brazil; 4 Faculty of Health Sciences, Lúrio University, Nampula, Mozambique; 5 Centre for Data and Knowledge Integration for Health, Oswaldo Cruz Foundation, Sl 315. Rua Mundo, 121. Trobogy, Salvador, Bahia 41745-715 Brazil; 6 Center On Racism, Global Movements, and Population Health Equity Drexel University Dornsife School of Public Health, Philadelphia, USA; 7 Institute of Collective Health, Federal University of Mato Grosso (UFMT), Cuiabá, MT Brasil; 8 Institute of Collective Health, Federal University of Bahia (UFBA), Salvador, Bahia Brazil; 9 Barcelona Institute for Global Health, Hospital Clinic, Universitat de Barcelona, Barcelona, Spain; 10 School of Nutrition, Federal University of Bahia (UFBA), Salvador, Bahia Brazil

**Keywords:** Low birth weight, Small for gestational age, Large for gestational age, Food environment, Maternal-infant health

## Abstract

**Introduction:**

Birth weight is described as one of the main determinants of newborns’ chances of survival. Among the associated causes, or risk factors, the mother’s nutritional status strongly influences fetal growth and birth weight outcomes of the concept. This study evaluates the association between food deserts, small for gestational age (SGA), large for gestational age (LGA) and low birth weight (LBW) newborns.

**Design:**

This is a cross-sectional population study, resulting from individual data from the Live Birth Information System (SINASC), and commune data from mapping food deserts (CAISAN) in Brazil. The newborn’s size was defined as follows: appropriate for gestational age (between 10 and 90^th^ percentile), SGA (< 10^th^ percentile), LGA (> 90^th^ percentile), and low birth weight < 2,500 g. To characterize food environments, we used tertiles of the density of establishments which sell *in natura* and ultra-processed foods. Logistic regression modeling was conducted to investigate the associations of interest.

**Results:**

We analyzed 2,632,314 live births in Brazil in 2016, after appropriate adjustments, women living in municipalities with limited availability of fresh foods had a higher chance of having newborns with SGA [OR2nd tertile: 1.06 (1.05–1.07)] and LBW [OR2nd tertile: 1.11 (1.09–1.12)]. Conversely, municipalities with greater availability of ultra-processed foods had a higher chance of having newborns with SGA [OR3rd tertile: 1.04 (1.02–1.06)] and LBW [OR2nd tertile: 1.13 (1.11–1.16)]. Stratification by race showed that Black and Mixed/Brown women had a higher chance of having newborns with SGA [OR3rd tertile: 1.09 (1.01–1.18)] and [OR3rd tertile: 1.06 (1.04–1.09)], respectively, while Mixed-race women also had a higher chance of having newborns with LBW [OR3rd tertile: 1.17 (1.14–1.20)]. Indigenous women were associated with LGA [OR3rd tertile: 1.20 (1.01–1.45)].

**Conclusion:**

The study found that living in areas with limited access to healthy foods was associated with an increased risk of SGA and low birth weight among newborns, particularly among Black and Mixed/Brown women. Therefore, urgent initiatives aimed at reducing social inequalities and mitigating the impact of poor food environments are needed in Brazil.

**Supplementary Information:**

The online version contains supplementary material available at 10.1186/s12884-023-05947-1.

## Introduction

Food deserts refer to areas where the availability of healthy foods, whether in quantity or quality, is lacking. These areas are typically found in low-income urban or rural communities [1]. In these communities, limited economic conditions often restrict access to healthy foods, leading to an increased reliance on processed and ultra-processed foods, which have been associated with excessive gestational weight gain and the birth of large for gestational age (LGA) babies [2–4].

The impact of the food environment on health and quality of life is significant, especially for the health of infants. A mother's diet during pregnancy plays a crucial role in ensuring the health and well-being of both the mother and the baby. Inadequate intake of essential vitamins and minerals, as well as high consumption of food and drinks with refined sugar and saturated fat, can increase the likelihood of low birth weight (LBW) babies. This can have long-term health consequences for the child [2]. In addition to the impact on the general population, food deserts also have a profound effect on the lives of babies, from their intrauterine growth to conception, and their subsequent growth and development [1]. Unhealthy foods that offer high energy density and low nutritional quality, especially processed and ultra-processed foods, contribute to excessive weight gain and the development of non-communicable chronic diseases (NCDs), such as diabetes, cardiovascular disease, cancer, among others [5].

Consuming a healthy and balanced diet is crucial, and this includes foods such as fruits, vegetables, whole grains, lean meats, fish, eggs, nuts, and seeds [6]. Ensuring proper nutrition is crucial during the pre-conceptional period, pregnancy, and breastfeeding, as it promotes the health of both the mother and the fetus. However, the majority of processed foods, such as snacks, sodas, fast food, and pre-packaged meals, tend to be less healthy and are associated with negative health outcomes. This is because many processed foods contain high levels of sugar, saturated fats, sodium, and artificial additives, which can contribute to health problems such as obesity, heart disease, and diabetes [7, 8]. When planning a maternal diet, it's important to keep in mind that inadequate intake of essential vitamins and minerals, as well as high consumption of food and drinks containing refined sugar and saturated fat, can increase the risk of having low birth weight (LBW) babies. In the twentieth century, newborns weighing less than 2,500 g were considered high-risk or LBW babies [9].

Maternal body composition during intrauterine growth may contribute to a greater risk of cardiometabolic disease in newborns who are small or large for gestational age (SGA and LGA). The compensations made for intrauterine growth, such as recovery or reduction of postnatal growth, may lead to adverse consequences [10]. Being born SGA is associated with increased infant morbidity and mortality rates, as well as alterations in growth patterns and body composition, which may be linked to the development of risk factors for metabolic and cardiovascular diseases [11]. Excessive gestational weight gain and the presence of metabolic comorbidities are among the maternal factors associated with the birth of LGA babies, while insufficient gestational weight gain is associated with the birth of SGA babies [12, 13]. Birth weight is a major determinant of newborn survival and has been significantly associated with various factors, such as the mother's level of education, the number of prenatal appointments, ethnicity/race, maternal weight during pregnancy [14, 15], and the presence of food [14, 16]The impact of food environment on public health, specifically on the association between these enviroment and the birth of SGA, LGA, and LBW babies, is a vital aspect that needs to be understood [17] Therefore, this study seeks to explore the association between the food environment and these outcomes.

## Methods

### Study design, population, and data source

This is a cross-sectional ecological study that utilizes secondary data obtained from the merging of two population studies. The first set of data was obtained through the Data Science Applied to Health Platform (PCDaS/Icict/Fiocruz) from the Ministry of Health Unified Health System, Department of Information and IT (DATASUS), specifically the 2016 Live Birth Information System (SINASC) [18]. The second set of data was produced by the Interministerial Chamber of Food and Nutritional Security (CAISAM) in 2016, which provided information on food deserts [19].

### Study variables

#### Outcome: fetal growth

Fetal growth was categorized as: appropriate for gestational age (between 10 and 90^th^ percentile), SGA (< 10^th^ percentile), or LGA (> 90^th^ percentile), using specific curves by sex corresponding to single live births, as established by the INTERGROWTH-21^st^ Consortium [20], to classify weight by gestational age (24/0 to 42/0 gestational weeks). The LBW outcome was considered from the dichotomous classification of the birth weight variable: LBW: < 2,500 g, and normal weight: ≥ 2,500 g.

The SINASC (Live Birth Information System) is an information system of the Brazilian Ministry of Health that collects data on births occurring in the country. The information is collected through the Declaration of Live Birth (DNV), filled out by healthcare professionals who assist in the delivery. The coverage of the SINASC is national and includes all births that occur in hospitals and maternity units, both public and private, as well as home births registered with the civil registry office. However, it is important to note that not all births are registered in the SINASC. There are cases where registration does not occur, such as in births that take place in locations without healthcare professionals, such as at home without assistance or in remote areas without access to healthcare services. According to data from the Ministry of Health, the average coverage of the SINASC in Brazil is about 97% [18].

#### Main independent variables

The main independent variable was the food environment. The exposure of pregnant women was organized based on the region in which the mothers resided, that is, by the commune in which they lived. This approach allows for the evaluation of the impact of the food environment on the diet of pregnant women, considering the nutritional characteristics of the foods available in the vicinity of their residences.

The information on the food environment was obtained from a study conducted by the Interministerial Chamber of Food and Nutritional Security (CAISAN) in 2018. The study aimed to map food deserts in Brazil using data from various sources. The Annual Social Information Report (RAIS-2016) was used to identify establishments classified by the National Classification of Economic Activities (CNAE). Additionally, data from the organic fair map of street markets produced by the Brazilian Institute for Consumer Defense (IDEC), SAN map of markets, and food markets listed on the electronic sites of city halls in Brazilian state capitals were incorporated. The Family Budget Research (POF 2008–2009) was used to identify the food categories purchased by the population and the respective locations of these purchases. The food acquired was then classified according to the four categories proposed by the 2014 Food Guide for the Brazilian Population (Ministry of Health, 2014). The establishments were categorized into three groups: those that predominantly sold fresh foods (over 50%), those that sold ultra-processed foods, and mixed establishments that did not have a predominant supply of either healthy or unhealthy foods. In the context of food environments, food establishments can be organized as follows: Restaurants, Cafeterias, Bakeries, Bars, Food courts, Markets, and Supermarkets [21, 22]. The term "in natura" refers to food that is in its natural state or minimally processed. It typically refers to fresh, whole foods that have undergone minimal or no processing, such as fruits, vegetables, whole grains, nuts, and seeds. These foods are generally considered healthier options as they retain their natural nutrients and are free from additives, preservatives, and artificial ingredients. On the other hand, ultra-processed food refers to food products that have undergone extensive processing and contain a high number of additives, preservatives, and artificial ingredients. These foods are typically made from refined ingredients and often have little nutritional value. Examples of ultra-processed foods include sugary drinks, packaged snacks, fast food, frozen meals, and processed meats. These foods are often high in added sugars, unhealthy fats, and sodium, and their consumption in excess is associated with various health issues, such as obesity, heart disease, and diabetes [7, 8].

To characterize the food environments, only the density of establishments selling in natura and ultra-processed foods per 10,000 inhabitants was used. These results were then divided into tertiles and used in the statistical models. We decided to use tertiles for analyzing the food environment for several reasons, such as simplicity, research context, adequate sample size, and robustness [23].

### Covariates

The covariates considered at the individual level were as follows: mother’s age (≤ 17; 18–19; 20–34; and ≥ 35); marital status (married, civil partnership, single, widow, or separated); the mother's level of education at time of birth (none; 1–7; 8–11; and 12 or more); gestational age in weeks (22–27, 28–31, 32–36, and 37–42 weeks), number of prenatal appointments (none, between 1 and 3, between 4 and 6, and 7 and more); newborn’s sex (male or female); and mother’s race/color (White; Black, Yellow (Asian descendant), Mixed/Brown, and indigenous).

The contextual variables include: (Gross Domestic Product (GDP) per capita, Gini index, unemployment rate, Family Health Strategy (ESF) coverage, commune Human Development Index obtained from IBGE 2000 and 2010 Demographic Census data, extrapolated for 2016, and provided by the IBGE SIDRA Automatic Recovery [24] system by the DATASUS TabNet tool [25], and the United Nations Development Programme (UNDP) Human Development Atlas in Brazil [26].

### Statistical analysis

The socioeconomic, maternal, and birth characteristics were summarized using frequency distributions. Logistic regression models were used to investigate factors associated with small for gestational age (SGA), large for gestational age (LGA), low birth weight (LBW), and food deserts, defined by the density of in natura and ultra-processed foods (in tertiles), which were the main independent variables. The models were simultaneously adjusted for covariates. Variables were stepwise included in the model, and those with a univariate p-value < 0.25 were tested. The magnitude was quantified using odds ratios (OR) with a 95% confidence interval (CI 95%). All analyses were conducted using available covariates considered plausible and relevant in the literature. To ensure adequate covariate adjustment, we used a stepwise adjustment strategy, including all relevant covariates and testing their influence on the model.

All analyses were performed using the available covariates that were deemed plausible and relevant in the literature [27–29]. To select the contextual variables and theoretical framework, a correlation analysis was performed to assess multicollinearity. A conceptual model was employed to introduce the variables, as shown in Supplementary Figure (Fig. S1). The analysis was carried out using R, version 3.6.1 (http://www.r-project.org), and RStudio software, version 1.2.1335.

## Results

Table 1 presents sociodemographic data for Brazil, including a GDP per capita of BRL 17,600 and an unemployment rate of approximately 4.6%. The Family Health Strategy (ESF), which is the primary basic healthcare policy in the country, had a coverage rate of 88.14%. For more details, please refer to the table.

**Table 1:** Demographic and socio-economic characteristics of Brazilian Communes, 2016

Variables	Average	Standard Deviations	Minimum	Median	Maximum
Commune HDI	0.74	0.58	0.51	0.74	0.85
Gini Index	0.47	0.92	0.16	0.47	0.92
GDP per capita	17.6	21.6	2.99	12.73	486.34
Unemployment rate (%)	4.56	4.42	0.00	3.39	58.63
ESF coverage (%)	88.14	22.23	0.00	100	100

**Fig. 1:**
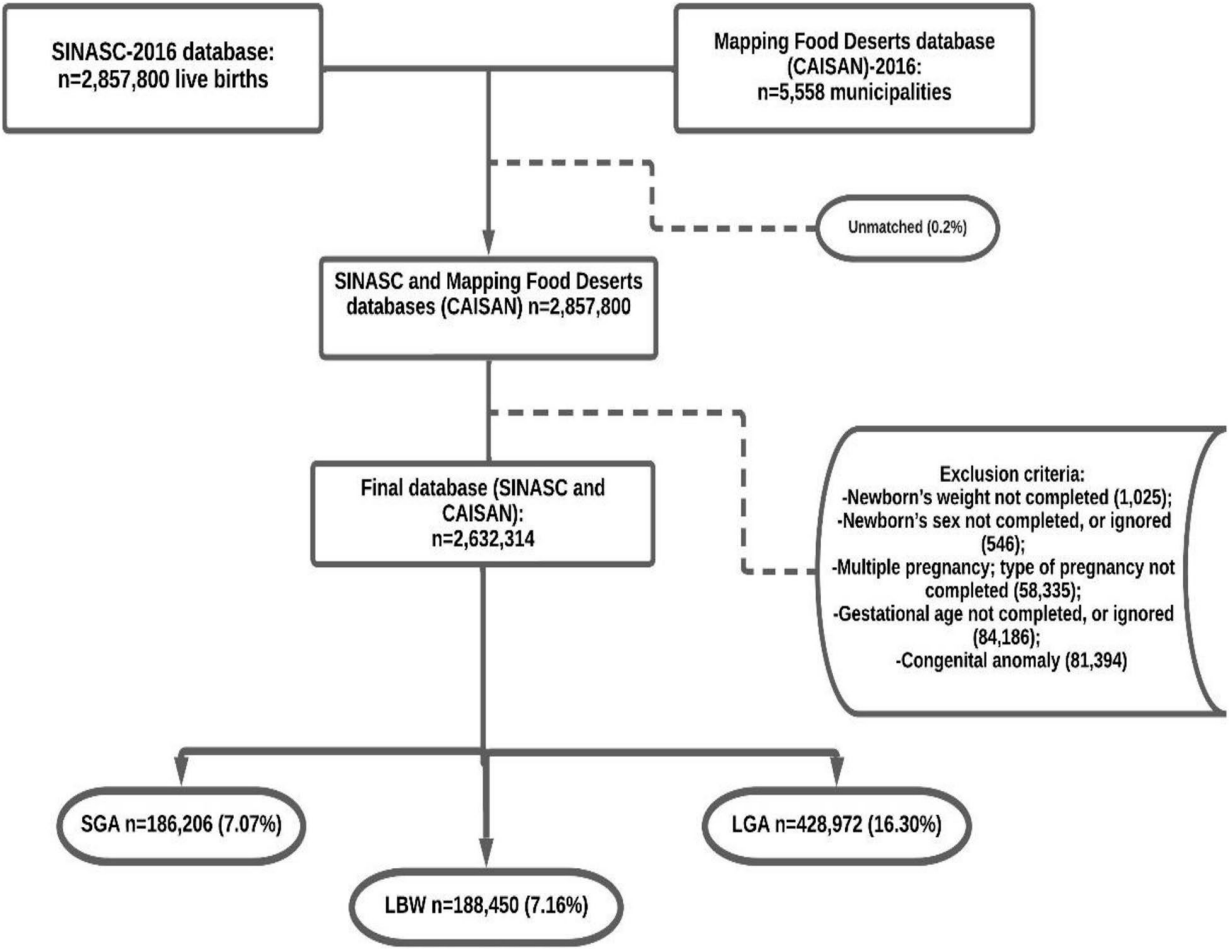
Study population

Figure 1 shows that the study included 2,632,314 live births, of which 186,206 (7.07%), 428,972 (16.30%), and 188,450 (7.16%) were classified as SGA, LGA, and LBW, respectively.

The study population characteristics reveal that SGA (small for gestational age) and LBW (low birth weight) are prevalent among women aged ≥ 35, with limited education, indigenous or black ethnicity, fewer prenatal appointments, and those who are single, widowed, or divorced. Moreover, female newborns appear to have a higher incidence of SGA and LBW (Table 2).

**Table 2:** Food environment, socio-economic, maternal, and live birth characteristics, in accordance with fetal growth and birth weight (*n* = 2,632,314) in 2016

Variables	Missing No (%)	n(%)	Fetal growth		LBW No (%)
			**SGA No (%)**	**LGA No (%)**	
**Density of unhealthy food**	1,499 (0.06)				
1st tertile (< p33.3)		877,391 (33.4)	69,258 (7.9)	158,519 (18.1)	59,216 (6.7)
2nd tertile (> = p33.3; = < p66.6)		888,127 (33.7)	60,703 (6.8)	141,863 (16.0)	64,965 (7.3)
3rd tertile (> p66.6)		865,297 (32.9)	56,117 (6.5)	128,333 (14.8)	64,165 (7.4)
**Density of *in natura* foods**	1,499 (0.06)				
1st tertile (< p33.3)		876,820 (33.3)	63,814 (7.3)	150,502 (17.2)	60,077 (6.9)
2nd tertile (> = p33.3; = < p66.6)		877,017 (33.5)	60,252 (6.9)	133,752 (15.3)	65,009 (7.4)
3rd tertile (> p66.6)		876,978 (33.2)	62,012 (7.1)	144,461 (16.5)	63,260 (7.2)
**Mother’s age (years)**	0 (0.0)				
20–34		990,917 (33.4)	186,036 (6.1)	990,917 (18.8)	71,386 (7.2)
≤ 17		1,377,950 (33.4)	108,608 (7.9)	197,87 (14.4)	99,857 (7.3)
18–19		255,561 (33.4)	16,258 (6.4)	43,648 (17.1)	16,229 (6.4)
≥ 35		7,886 (33.4)	760 (9.7)	1,418 (18.0)	978 (12.4)
**Gestational age in weeks**	0 (0.0)				
22–27		9,043 (0.34)	930 (10.3)	2,869 (31.7)	7,766 (85.9)
28–31		23,450 (0.89)	2,598 (11.1)	9,042 (38.6)	17,900 (76.3)
32–36		236,374 (8.98)	14,711 (6.2)	75,493 (31.9)	82,508 (34.9)
37–42		2,363,447 (89.8)	167,967 (7.1)	341,568 (14.5)	80,276 (3.4)
**Marital status**	25,342 (0.1)				
Married, civil partnership		1,456,118 (55.9)	94,021 (6.5)	249,27 (17.1)	96,530 (6.6)
Single, widow, or separated		1,150,854 (44.1)	90,215 (7.8)	175,278 (15.2)	90,079 (7.8)
**Mother’s level of education**	31,214 (1.2)				
More than 12 years		503,279 (19.3)	81,335 (5.1)	503,279 (16.6)	32,931 (6.5)
None		12,146 (0.5)	1,640 (13.5)	2,165 (17.8)	1,186 (9.8)
1 to 3 years		59,845 (2.3)	5,848 (9.7)	11,344 (19.0)	5,058 (8.4)
4 to 7 years		449,521 (17.3)	39,232 (8.7)	74,904 (16.7)	35,834 (8.0)
8 to 11 years		1,576,309 (60.6)	111,222 (7.1)	253,604 (16.1)	111,255 (7.06)
**Number of prenatal appointments**	13,496 (0.5)				
0.5		1,805,975(68.9)	114,922 (6.4)	293,926 (16.2)	91,113 (5.0)
None		41,253 (1.6)	4,798 (11.6)	5,809 (14.1)	6,251 (15.2)
1 to 3		156,307 (6.0)	14,792 (9.5)	26,137 (16.7)	22,886 (14.6)
4 to 6		615,283 (23.5)	50,327 (8.2)	101,100 (16.4)	66,207 (10.8)
**Newborn’s sex**	0 (0.0)				
Male		1,347,479 (51.2)	93,028 (6.9)	221,601 (16.4)	88,060 (6.5)
Female		1,284,835 (48.8)	93,178 (7.3)	207,371 (16.4)	100,390 (7.8)
**Mother’s race/ethnicity**	98,309 (3.7)				
White		945,556 (37.3)	56,112 (5.9)	148,12 (15.7)	65,985 (7.0)
Black		136,575 (5.4)	11,678 (8.6)	20,841 (15.2)	11,879 (8.7)
Yellow		9,496 (0.4)	638 (6.7)	1,335 (14.1)	725 (7.6)
Mixed-race		1,421,330 (56.1)	108,048 (7.6)	238,343 (16.8)	100,815 (7.1)
Indigenous		21,048 (0.8)	2,477 (11.7)	3,945 (18.7)	1,569 (7.45)

Table 3 The results of the adjusted binary logistic regression to assess the relationship between densities of processed and in natura foods and birth weight are presented. After making necessary adjustments, it was observed that women from commune with limited availability of in natura foods had a higher likelihood of having a small for gestational age (SGA) newborn [odds ratio (OR) for 2nd tertile: 1.06 (1.05–1.07)], and a low birth weight (LBW) [OR for 1st tertile: 1.10 (1.08–1.12), OR for 2nd tertile: 1.11 (1.09–1.12) with a dose–response effect]. Stratification demonstrated that being of mixed/brown-race was associated with SGA [OR for 1st tertile: 1.02 (1.01–1.03), OR for 2nd tertile: 1.07 (1.06–1.10) with a dose–response effect], LBW [OR for 1st tertile: 1.13 (1.12–1.16), OR for 2nd tertile: 1.12 (1.10–1.14)], and black with LBW [OR for 1st tertile: 1.14 (1.07–1.22)].

**Table 3:** Adjusted Odds Ratio (OR) and 95% confidence interval for the association between food density and SGA, LGA, and LBW in binary logistic regression and models stratified by race (*n* = 2,632,314)

	**Food environments**			
	***in natura* food density**		**Ultra-processed food density**	
**Variables**	**2nd tertile (> = p33.3; = < p66.6)**	**1st tertile (< p33.3)**	**2nd tertile (> = p33.3; = < p66.6)**	**3rd tertile (> p66.6)**
	**Adjusted OR (CI 95%)**	**Adjusted OR (CI 95%)**	**Adjusted OR (CI 95%)**	**Adjusted OR (CI 95%)**
**SGA**	**1.06 (1.05–1.07)**	**1.01 (1.01–1.03)**	0.99 (0.98–1.01)	**1.04 (1.02–1.06)**
**LGA**	0.93 (0.92–0.94)	0.97 (0.96–0.98)	0.93 (0.92–0.94)	0.87 (0.85–0.88)
**LBW**	**1.11 (1.09–1.12)**	**1.10 (1.08–1.12)**	**1.11 (1.09–1.13)**	**1.13 (1.11–1.16)**
**SGA**				
White	1.02 (0.99–1.04)	0.98 (0.96–1.01)	0.96 (0.93–0.99)	0.97 (0.93–1.01)
Black	1.05 (0.99–1.11)	1.01 (0.99–1.07)	1.02(0.96–1.09)	**1.09 (1.01–1.18)**
Yellow	0.95 (0.77–1.18)	0.71 (0.57–0.89)	0.91 (0.69–1.19)	1.11 (0.81–1.51)
Mixed-race	**1.07 (1.06–1.10)**	**1.02 (1.01–1.03)**	0.98 (0.96–0.99)	**1.06 (1.04–1.09)**
Indigenous	1.00 (0.89–1.12)	0.89 (0.78–1.03)	1.03 (0.86–1.23)	0.94 (0.71–1.24)
**LGA**				
White	0.99 (0.97–1.01)	1.03 (0.98–1.05)	0.94 (0.92–0.96)	0.91 (0.89–0.93)
Black	0.89 (0.85–0.93)	0.95 (0.91–0.98)	0.94 (0.89–0.99)	0.90 (0.85–0.95)
Yellow	0.87 (0.74–1.02)	0.92 (0.78–1.08)	1.04 (0.85–1.05)	0.95 (0.75–1.20)
Mixed-race	0.89 (0.88–0.90)	0.95 (0.94–0.96)	0.94 (0.93–0.95)	0.82 (0.81–0.84)
Indigenous	1.02 (0.93–1.12)	0.91 (0.82–1.03)	0.97 (0.84–1.11)	**1.20 (1.01–1.45)**
**LBW**				
White	1.04 (0.96–1.07)	1.01 (0.99–1.05)	0.99 (0.97–1.14)	1.01 (1.00–1.14)
Black	**1.14 (1.07–1.22)**	**1.14 (1.08–1.22)**	1.01 (0.93–1.09)	1.07 (0.98–1.17)
Yellow	1.19 (0.95–1.51)	0.86 (0.66–1.11)	0.83 (0.62–1.13)	1.04 (0.74–1.47)
Mixed-race	**1.13 (1.12–1.16)**	**1.12 (1.10–1.14)**	**1.09 (1.07–1.12)**	**1.17 (1.14–1.20)**
Indigenous	0.98 (0.86–1.13)	1.17 (0.99–1.34)	1.22 (0.99–1.48)	0.99 (0.74–1.33)

*in natura* food density: Ref. 3rd tertile; Ultra-processed food density: Ref. 1st tertile

Analyses adjusted by: Mother’s age; marital status, mothers’ level of education by years of study; gestational age in weeks, number of prenatal appointments; newborn’s sex; race/color; HDI (human development index); GDP per capita; unemployment rate; Gini index and ESF coverage (%)

Additionally, communes with a higher density of ultra-processed foods had greater odds of having SGA [OR for 3rd tertile: 1.04 (1.02–1.06)] and LBW newborns [OR for 1st tertile: 1.11 (1.09–1.13), OR for 2nd tertile: 1.13 (1.11–1.16) with a dose–response effect]. Stratification revealed that black and mixed-race women, respectively, were associated with SGA [OR for 3rd tertile: 1.09 (1.01–1.18)] and [OR for 3rd tertile: 1.06 (1.04–1.09)]. Only mixed-race women were associated with LBW [OR for 2nd tertile: 1.09 (1.07–1.12), OR for 3rd tertile: 1.17 (1.14–1.20)]. Being indigenous was associated with a large for gestational age (LGA) [OR for 3rd tertile: 1.20 (1.01–1.45)]. The raw analyses are available in the supplementary tables (Table S1).

## Discussion

The study aimed to investigate the relationship between food environments, fetal growth, and birth weight in Brazil. The results indicated that there is a higher likelihood of women giving birth to babies with low birth weight and small for gestational age in communes with higher availability of ultra-processed foods and low availability of fresh foods. This pattern was more evident among black and mixed-race women. In contrast, indigenous women living in areas with a high density of ultra-processed foods had a higher chance of having newborns large for gestational age. These results support the position that the density of ultra-processed foods may play a greater role than the density of fresh foods in fetal growth and birth weight [28].

The present study addresses the relationship between the food environment and fetal growth, which is still a controversial topic. However, the results presented support the existing literature, which indicates that these environments can amplify the effects of an inadequate diet, compromising perinatal and neonatal outcomes [16, 28, 30–33]. A similar study conducted by Sawangkum et al. (2020) revealed that women living in desert areas with poor food access were associated with newborns with low birth weight. Lack of access to food was also associated with small-for-gestational-age births after adjusting for maternal race/ethnicity [16]. Additionally, the research conducted by Saeed and colleagues, which aimed to evaluate the effect of maternal food insecurity on birth weight, also demonstrated that women who experienced food insecurity had a higher risk of having a newborn with low birth weight [32]. There are several studies that confirm the relationship between limited access to food and the birth of low-weight babies. A study conducted with 8,753 households also showed that the chance of having low birth weight babies was higher for those in food insecurity situations, for female infants, and for mothers who had attended fewer prenatal visits [34]. Similarly, mothers experiencing food insecurity had increased chances of giving birth to a low birth weight baby, especially among girls [35]. A different result regarding ultra-processed foods was observed in a study where the percentage of energy intake from ultra-processed foods was a predictor of increased neonatal body fat [36]. This may explain the finding in indigenous women residing in communes with higher density of ultra-processed foods and higher likelihood of having large babies for gestational age. Previously, this had been associated with the invasion of the food industry in more rural areas, changing the population's consumption profile and the lack of alternatives to access healthier food options. It is well known that the food industry mediates the increase in calories and consequent excessive gestational weight gain [37–40]. The increase in access to supermarkets is associated with a lower prevalence of overweight and obesity, as well as an improvement in fruit and vegetable consumption in pregnant women28 [33].

On the other hand, Richterman et al. [28] found that food insecurity is a risk factor for the birth of small-for-gestational-age infants. Women with low gestational weight gain who resided in food deserts were at high risk of premature delivery, along with other factors such as age, maternal race, education level, marital status, and interpregnancy intervals [40]. Additionally, an ecological study showed that women living in neighborhoods with food insecurity had a higher chance of giving birth to very small-for-gestational-age infants [41].

Black women residing in areas with limited access to natural foods have a higher risk of giving birth to low birth weight or premature newborns [42]. Risk factors and unfavorable outcomes associated with limited access to healthy food were more prevalent in areas with medium and high concentrations of Black people [43, 44]. Therefore, it is evident that social and health inequalities related to racial characteristics of vulnerable and minority social groups should be considered in public policies for social protection. It is important to emphasize that the strong association with unfavorable outcomes for being Black, mixed-race, and Indigenous can be observed in the results of this study.

The main limitation of this study arises from the impossibility of attaining exposure (food density) in an individualized way to gain a better understanding of the context. However, the methodological design provided a practical approach to evaluate the objective of this study. Another possible limitation is the quality and reliability of secondary data, which may introduce biases related to missing values, under-estimation, and classification errors. In this respect, data was obtained from government sources, such as health information systems and the Ministry of Citizenship, which are known for holding high quality standards. There may also be some unobserved variables that confuse the association we have studied, such as the absence of variables on the mother’s nutritional status, and gains in gestational weight, or diet, for example, which are significant confounders for the topic studied. One additional limitation of this study is the large sample size, which may increase the risk of Type I error.

## Conclusion

The study revealed that women residing in communes with a higher supply of ultra-processed foods and limited availability of natural foods, particularly Black and mixed-race women, have a greater likelihood of giving birth to low birth weight (LBW) and small-for-gestational-age (SGA) babies. Conversely, indigenous women living in areas with high densities of ultra-processed foods have a higher likelihood of giving birth to large-for-gestational-age (LGA) babies due to the excess supply of unhealthy foods that have low nutritional quality and come from unsustainable food systems. Therefore, urgent action is necessary to establish fairer and more egalitarian social policies that ensure access to healthy foods, a fundamental human right, and minimize the adverse perinatal and neonatal effects. Providing timely food supplements through social assistance should also be considered to help reduce perinatal and neonatal morbimortality. Additionally, policies promoting the production of agroecological and organic foods, and using economic and fiscal measures such as taxing food products high in saturated fat, sugar, and salt, should be implemented. Encouraging food and nutritional education activities based on food guides for pregnant women is also crucial to support and foster healthy habits. Future research is needed to understand this relationship and its impact on other maternal-infant outcomes.

### Supplementary Information

**Additional file 1:** **Table S1.** Raw Odds Ratio (OR) and 95% confidence interval for the association between food densities and SGG, LGA, and LBW in binary logistic regression models and models stratified by race (*n *=2,632,314).

**Additional file 2:** **Table S2.** Characterization of establishments which provide food services, or sell food, by commune size and type of establishment, Brazil. 2016 (*n *=5,558).

**Additional file 3:** **Fig. S1.** Conceptual model to investigate food environments as determinants of fetal growth and birth weight.

## Data Availability

The data used for this study was obtained from two sources. The first set of data was obtained through the Data Science Applied to Health Platform (PCDaS/Icict/Fiocruz) from the Ministry of Health Unified Health System, Department of Information and IT (DATASUS), available at: https://pcdas.icict.fiocruz.br/conjunto-de-dados/sistema-de-informacao-sobre-nascidos-vivos/. The second set of data was obtained from the Interministerial Chamber of Food and Nutritional Security (CAISAN) at the link: https://aplicacoes.mds.gov.br/sagirmps/portal-san/artigo.php?link=23.
