# Supplementary material for: Association between food environments and fetal growth in pregnant Brazilian women
Source: BMC Pregnancy Childbirth. 2023 Sep 13;23:661. doi: 10.1186/s12884-023-05947-1 (PMC10500732; doi:10.1186/s12884-023-05947-1)
Supplement: Supplementary file 1 — Additional file 1: Table S1. Raw Odds Ratio (OR) and 95% confidence interval for the association between food densities and SGG, LGA, and LBW in binary logistic regression models and models stratified by race (n =2,632,314). [file 12884_2023_5947_MOESM1_ESM.docx]

Table S1 – Raw Odds Ratio (OR) and 95% confidence interval for the association between food densities and SGG, LGA, and LBW in binary logistic regression models and models stratified by race (n=2,632,314).

|  | **Food environments** | | | |
| --- | --- | --- | --- | --- |
| **Variables** | *in natura* **food density** | | **Ultra-processed food density** | |
|  | **2nd tertile (>=p33.3; =<p66.6)** | **1st tertile (<p33.3)** | **2nd tertile (>=p33.3; =<p66.6)** | **3rd tertile (>p66.6)** |
|  | **Adjusted OR (CI 95%)** | **Adjusted OR (CI 95%)** | **Adjusted OR (CI 95%)** | **Adjusted OR (CI 95%)** |
| **SGA** | 0.94 (0.93-0.95) | 0.97 (0.96-0.98) | 1.02 (1.01-1.05) | 1.03 (1.05-1.08) |
| **LGA** | 0.87 (0.86-0.88) | 0.95 (0.95-0.97) | 0.86 (0.86-0.88) | 0.79 (0.78-0.80) |
| **LBW** | 1.08 (1.07-1.10) | 1.06 (1.04-1.07) | 1.09 (1.08-1.10) | 1.10 (1.09-1.12) |
| **SGA** |  |  |  |  |
| White | 0.98 (0.96-1.00) | 0.99 (0.97-1.02) | 0.89 (0.86-0.91) | 0.85 (0.83-0.87) |
| Black | 1.04 (0.99-1.10) | 1.01 (0.96-1.06) | 0.99 (0.94-1.04) | 1.03 (0.98-1.09) |
| Yellow | 0.71 (0.60-0.86) | 0.69 (0.56-0.86) | 0.75 (0.69-0.93) | 0.73 (0.60-0.88) |
| Mixed-race | 1.03 (1.01-1.04) | 1.01 (0.99-1.03) | 0.90 (0.89-0.92) | 0.92 (0.91-0.94) |
| Indigenous | 1.02 (0.92-1.12) | 0.77 (0.68-0.88) | 0.55 (0.48-0.64) | 0.48 (0.39-0.59) |
| **LGA** |  |  |  |  |
| White | 0.94 (0.93-0.96) | 1.01 (0.99-1.02) | 0.84 (0.86-0.90) | 0.88 (0.83-0.86) |
| Black | 0.83 (0.80-0.87) | 0.93 (0.90-0.97) | 0.80 (0.77-0.83) | 0.73 (0.71-0.76) |
| Yellow | 0.81 (0.71-0.93) | 0.97 (0.83-1.13) | 0.91 (0.78-1.07) | 0.76 (0.66-0.89) |
| Mixed-race | 0.85 (0.84-0.86) | 0.95 (0.94-0.96) | 0.87 (0.85-0.88) | 0.75 (0.74-0.76) |
| Indigenous | 0.97 (0.89-1.05) | 0.96 (0.88-1.06) | 1.09 (0.98-1.20) | 1.22 (1.07-1.39) |
| **LBW** |  |  |  |  |
| White | 1.01 (0.96-1.08) | 1.00 (0.99-1.06) | 0.91 (0.89-1.09) | 0.99 (0.95-1.10) |
| Black | 1.14 (1.07-1.20) | 1.12 (1.06-1.17) | 1.09 (1.04-1.15) | 1.17 (1.11-1.23) |
| Yellow | 0.86 (0.72-1.02) | 0.80 (0.64-0.97) | 0.86 (0.69-1.06) | 0.90 (0.74-1.08) |
| Mixed-race | 1.15 (1.13-1.17) | 1.08 (1.17-1.10) | 1.11 (1.10-1.13) | 1.19 (1.17-1.21) |
| Indigenous | 1.00 (0.89-1.13) | 1.01 (0.88-1.17) | 0.99 (0.86-1.15) | 0.74 (0.59-0.92) |
