# Supplementary material for: Association between food environments and fetal growth in pregnant Brazilian women
Source: BMC Pregnancy Childbirth. 2023 Sep 13;23:661. doi: 10.1186/s12884-023-05947-1 (PMC10500732; doi:10.1186/s12884-023-05947-1)
Supplement: Supplementary file 2 — Additional file 2: Table S2. Characterization of establishments which provide food services, or sell food, by commune size and type of establishment, Brazil. 2016 (n =5,558). [file 12884_2023_5947_MOESM2_ESM.docx]

Table S.2 – Characterization of establishments which provide food services, or sell food, by commune size and type of establishment, Brazil. 2016 (n=5,558).

| Municipality size |  |  |  |  | Type of establishment | |  |  |  |
| --- | --- | --- | --- | --- | --- | --- | --- | --- | --- |
|  | commune | *In natura* |  | Mixed |  | Ultra-processed | | Total | |
|  | N | N | % | N | % | N | % | N | % |
| Small | 4,952 | 42,691 | 19.28 | 132,401 | 59.79 | 46,345 | 20.93 | 221,437 | 100 |
| Medium | 327 | 15,043 | 17.90 | 48,042 | 57.17 | 20,945 | 24.93 | 84,030 | 100 |
| Large | 279 | 49,689 | 12.10 | 233,882 | 56.97 | 126,945 | 30.92 | 410,516 | 100 |
| Brazil | 5,558 | 107,423 | 15.00 | 414,325 | 57.87 | 194,235 | 27.13 | 715,983 | 100 |
