# Supplementary material for: Association between food environments and fetal growth in pregnant Brazilian women
Source: BMC Pregnancy Childbirth. 2023 Sep 13;23:661. doi: 10.1186/s12884-023-05947-1 (PMC10500732; doi:10.1186/s12884-023-05947-1)
Supplement: Supplementary file 3 — Additional file 3: Fig. S1. Conceptual model to investigate food environments as determinants of fetal growth and birth weight. [file 12884_2023_5947_MOESM3_ESM.pdf]

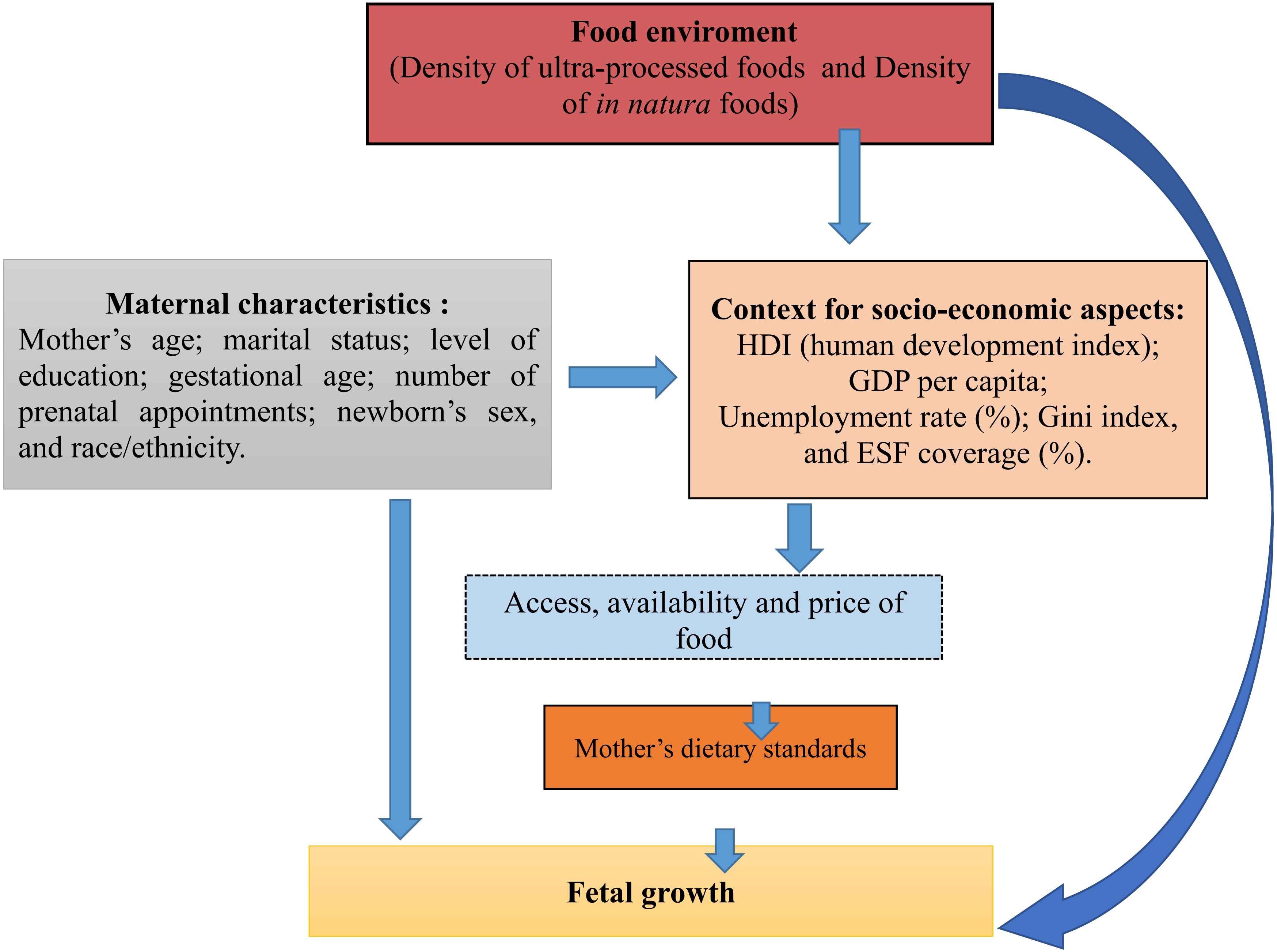

Fig S.1- **Conceptual model to investigate food environments as determinants of fetal growth and birth weight.**
